# Late antibody-mediated rejection after ABO-incompatible kidney transplantation during Gram-negative sepsis

**DOI:** 10.1186/1471-2369-15-31

**Published:** 2014-02-12

**Authors:** Annelies de Weerd, Alieke Vonk, Hans van der Hoek, Marian van Groningen, Willem Weimar, Michiel Betjes, Madelon van Agteren

**Affiliations:** 1 Erasmus Medical Center Rotterdam, Department of Nephrology, Room D-411, P.O. Box 2040, 3000 CA Rotterdam, The Netherlands; 2 Erasmus Medical Center Rotterdam, Department of Microbiology and Infectious diseases, Rotterdam, The Netherlands; 3 Erasmus Medical Center Rotterdam, Department of Hematology, Rotterdam, The Netherlands; 4 Erasmus Medical Center Rotterdam, Department of Pathology, Rotterdam, The Netherlands

**Keywords:** ABO-incompatible kidney transplantation, *Serratia marcescens*, Antibody-mediated rejection, Bacteremia-induced anti-ABO antibodies

## Abstract

**Background:**

The major challenge in ABO-incompatible transplantation is to minimize antibody-mediated rejection. Effective reduction of the anti-ABO blood group antibodies at the time of transplantation has made ABO-incompatible kidney transplantation a growing practice in our hospital and in centers worldwide. ABO antibodies result from contact with A- and B-like antigens in the intestines via nutrients and bacteria. We demonstrate a patient with fulminant antibody-mediated rejection late after ABO-incompatible kidney transplantation, whose anti-A antibody titers rose dramatically following *Serratia marcescens* sepsis.

**Case presentation:**

A 58-year-old woman underwent an ABO-incompatible kidney transplantation for end-stage renal disease secondary to autosomal dominant polycystic kidney disease. It concerned a blood group A1 to O donation. Pre-desensitization titers were 64 for anti-blood group A IgM and 32 for anti-blood group A IgG titers. Desensitization treatment consisted of rituximab, tacrolimus, mycophenolate mofetil, corticosteroids, immunoadsorption and intravenous immunoglobulines. She was readmitted to our hospital 11 weeks after transplantation for *S. marcescens* urosepsis*.* Her anti-A IgM titer rose to >5000 and she developed a fulminant antibody-mediated rejection.

We hypothesized that the (overwhelming) presence in the blood of *S. marcescens* stimulated anti-A antibody formation, as *S. marcescens* might share epitopes with blood group A antigen. Unfortunately we could not demonstrate interaction between blood group A and *S. marcescens* in incubation experiments.

**Conclusion:**

Two features of this post-transplant course are remarkably different from other reports of acute rejection in ABO-incompatible kidney transplantation: first, the late occurrence 12 weeks after kidney transplantation and second, the very high anti-A IgM titers (>5000), suggesting recent boosting of anti-A antibody formation by *S. marcescens*.

## Background

Both HLA and ABO blood group system determine the risk of rejection in clinical organ transplantation. The major challenge in blood group ABO-incompatible (ABO-i) transplantation is to minimize antibody-mediated rejection (AMR). In recent years, ABO-i kidney transplantation programs have been developed that minimize the risk for AMR and show excellent graft survival. The key to success has been effective reduction of the ABO antibodies prior to transplantation. This is usually achieved by repeated plasmapheresis with or without the use of a specific immunoadsorption procedure. A low concentration of ABO antibodies creates a window of opportunity for graft acceptance by an incompletely understood immunological phenomenon called “accommodation” [1,2]. Within the first week after transplantation, AMR may occur but these can usually be effectively reversed by current standard AMR treatment protocols. Anti-ABO titers usually remain low after transplantation. ABO antibodies are traditionally referred to as ‘natural occurring’ , since these antibodies were thought to occur without prior immunization. For over more than half a century, evidence is mounting that ABO antibodies most likely result from contact with A- and B-like antigens in the intestines via nutrients and bacteria, and develop early in childhood [3,4]. Therefore, boosting of ABO antibody titers may occur by infections with Gram-negative bacteria [5] and could, at least theoretically, cause AMR of ABO-i kidney transplants [6,7]. We present a case of a late fulminant AMR of an ABO-i kidney transplant which may have been triggered by Gram-negative bacteremia.

## Case presentation

A 58-year-old woman underwent living unrelated ABO-i kidney transplantation. Her medical history revealed hypertension and autosomal dominant polycystic kidney disease, for which she had been on peritoneal dialysis. She had never been pregnant and never received any blood products.

The donor kidney came from her 59-year-old husband and the HLA mismatch was 1-2-2 on A, B and DR loci respectively. She had no current or historical panel reactive antibodies. The donor blood group was A1 and recipient’s O. ABO desensitization treatment consisted of rituximab 375 mg/m^2^ 4 weeks before transplantation; tacrolimus 0.1 mg/kg BID, mycophenolate mofetil 1000 mg BID; prednisone 20 mg once daily starting two weeks before transplantation and immunoglobulines 0.5 mg/kg one day preoperatively. Five plasmapheresis sessions, followed by adsorption of anti-A antibodies with the Glycosorb® device coated with synthetically derived blood group A antigen, were performed in the week before transplantation. Her anti-A titer was 64 (IgM) and 32 (IgG) before treatment and decreased to 2 at the day before kidney transplantation. The surgical procedure was complicated by peri-transplant hematoma, for which erythrocyte concentrate and platelet transfusions were given (of blood group O donors). Immunosuppressive therapy after transplantation consisted of tacrolimus 4 mg BID, mycophenolate mofetil 1000 mg BID and prednisone 20 mg once daily. Valgancyclovir for cytomegolovirus (CMV) prophylaxis was started post-transplantation. Direct graft function was noted.

During admission, our patient experienced transient diarrhea and was treated for urinary tract infection with ciprofloxacin. Urine cultured *Pseudomonas aeruginosa* and *Serratia (S.) marcescens* (both > 10^5^ colony forming units (cfu)). Before discharge, a routine biopsy on day 14 revealed normal renal parenchyma, with no signs of rejection. Staining for C4d on endothelial cells was positive, which is often seen after ABO-i kidney transplantation and by itself does not indicate rejection. Anti-A titers remained low: one day post-operative the IgG titer was 2 and the IgM titer 8; at discharge, IgM titers were 1 and IgG titers were < 2. Renal function improved to a serum creatinine of 113 μmol/l at time of hospital discharge.

Seven weeks post-transplantation, patient was readmitted for fever and loose stools. She had developed new onset diabetes mellitus, for which intravenous insulin was started. Abdominal ultrasound revealed a swollen transplant with signs of pyelonephritis with multiple micro-abscesses. A 10-day course of ceftazidime and ciprofloxacin was started for suspected pyelonephritis as the urine culture identified various uropathogens, not further specified.

**Figure 1:**
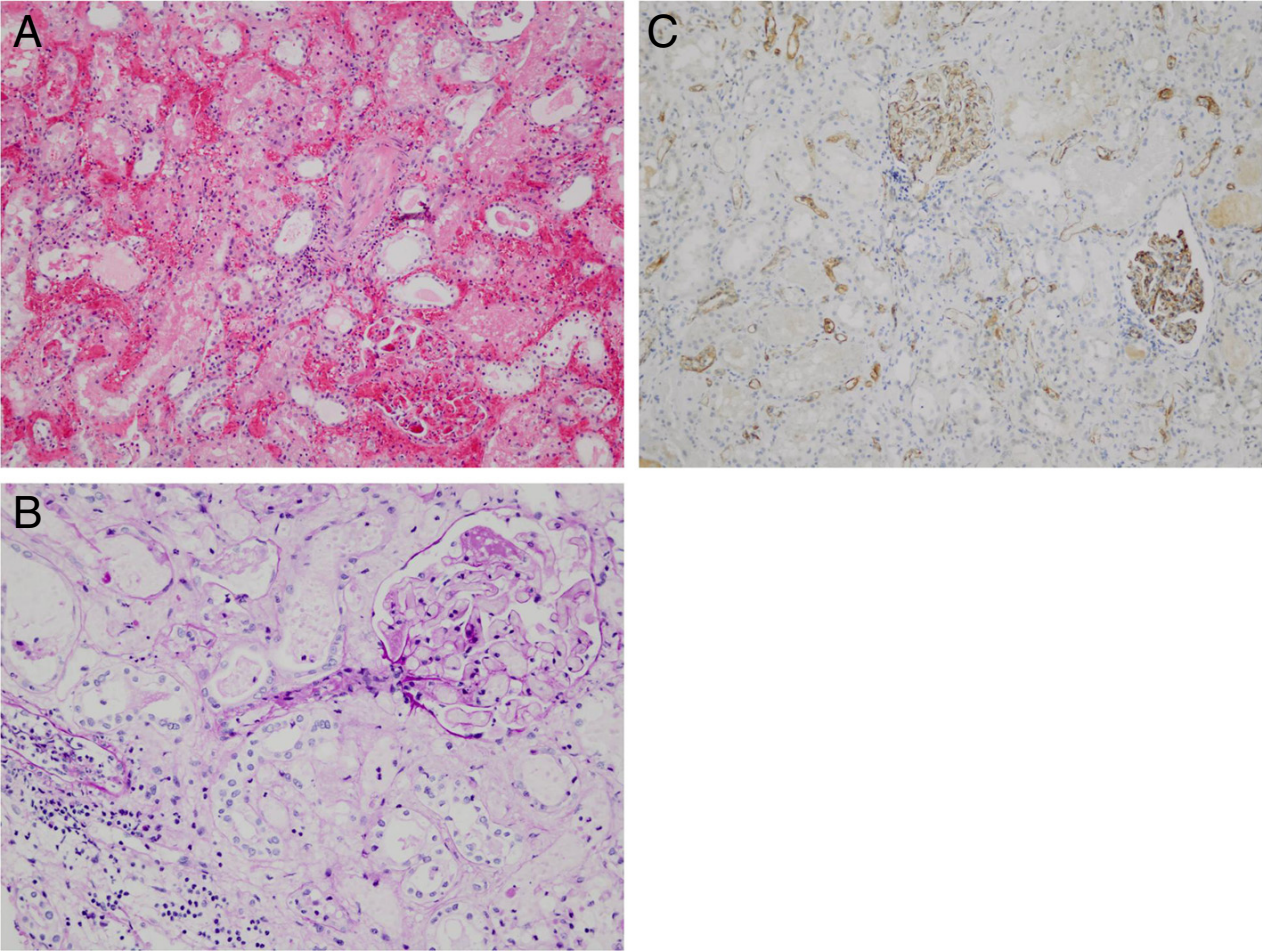
**Kidney transplant biopsy 12 weeks after ABO-incompatible kidney transplantation. A**. Severe hemorrhage of the cortex and congestion of the glomeruli and tubulointerstitial compartment, with only minimal influx of inflammatory cells. There is a thrombus in the arteriole of the glomerulus. (H&E staining; original magnification 10×). **B**. Congestion of the glomerulus with fibrinoid necrosis of the arteriole. There is ischemia of the tubuli. An artery shows a transmural inflammation, of both mononuclear cells and neutrophiles. (Periodic acid-Schiff-Diastase stain; original magnification 20×) **C**. Positive staining of more than 50% of the peritubular capillaries and all the glomeruli. (Immunohistochemistry for C4d; original magnification 10×).

**Figure 2:**
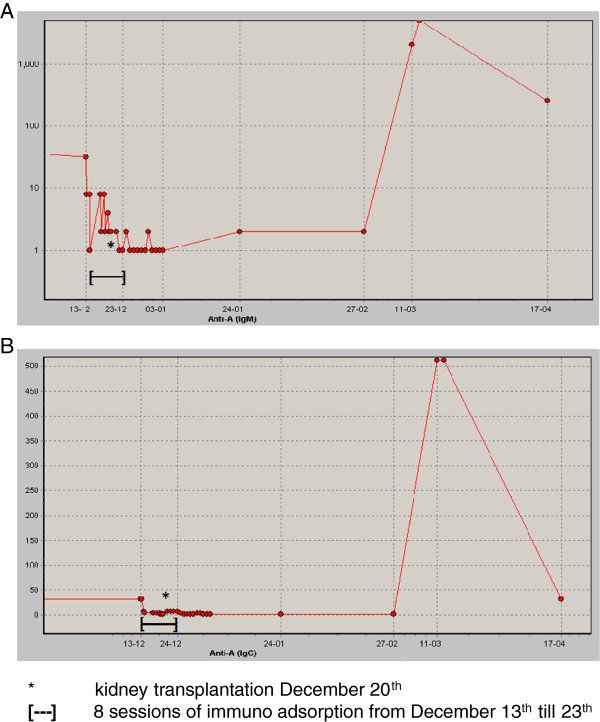
**Course of anti-A antibody titers before and after ABO-incompatible kidney transplantation.** The anti-A IgM **(A)** and IgG **(B)** titers were 64 and 32 respectively before pre-operative immunoadsorption (December 13th), decreased to 2/2 pre-operatively (December 20th) and were 1/<2 at discharge. During AMR they increased to >5000/512, decreasing to 256/32 one month later (logarithmic scale).

Eleven weeks post transplantation, patient returned to our emergency department with fever, tachycardia and pain over the renal allograft. Serum creatinine had risen to 115 umol/l with a C-reactive protein of 163 mg/l. Ultrasonography of the transplant kidney showed no gross abnormalities with normal renal vascular flow. Cultures of blood, urine and sputum were drawn and imipenem/cilastatine therapy was initiated. Only the blood culture became positive for *S. marcescens* sensitive to imipenem. In the next 5 days, serum creatinine increased further to 275 umol/l in combination with severe fluid retention. A newly obtained transplant ultrasound disclosed non-measurable diastolic blood flow. On the clinical suspicion of rejection, a three-day-course of methylprednisolone 1000 milligram intravenous was initiated and a transplant biopsy was performed. The kidney biopsy revealed AMR type 3 Banff ’09, with extended hemorrhagic infarction and positive C4d staining (Figure 1) [8]. The anti-A IgM titer was >5000 and anti-A IgG titer 512. Transplantectomy was performed as a renal scintigraphy showed no perfusion. A swollen and hemorrhagic kidney transplant was removed and chronic intermittent hemodialysis was initiated. A repeated anti-A titer one month later was 256 for IgM and 32 for IgG (Figure 2).

### Experiments

We hypothesized that the (overwhelming) presence in the blood of *S. marcescens* stimulated anti-A antibody formation, as *S. marcescens* might share epitopes with blood group A antigen. We chose to perform a hemagglutination inhibition assay instead of direct (serum) agglutination with bacteria, as the latter could occur because of possible aspecific clotting. *S. marcescens* obtained from the blood of our patient was frozen and stored until use. The thawed sample was plated on a (blood group free) Trypticase Soy agar (Becton Dickinson, USA) and grown at 37°C overnight. Cultures were suspended in phosphate buffered saline (PBS) and the concentration of bacteria in suspension was assessed using McFarland standards and plate counting the following day. Bacterial suspensions with concentrations ranging from 10^5^ cfu/ml to 10^8^ cfu/ml PBS and a PBS control were then either kept at 4 degrees Celsius, boiled, or sonicated. Subsequently, these different *S. marcescens* suspensions were incubated with anti-A plasma for 30 minutes at 37°C. Next, blood group A erythrocytes 4% (Sanquin blood supply, The Netherlands) were added and subsequently incubated for 15 minutes at room temperature. A PBS control was added to confirm visual agglutination after addition of A erythrocytes. We hypothesized that preincubation with a certain ‘threshold’ amount of bacteria would prevent hemagglutination. We extrapolated this experimental design from the methods of Springer et al. who demonstrated interaction between Gram-negative bacteria and anti-ABO antibodies in 1961 [9] (see Discussion for more details). Antibody titer changes were investigated as well: after centrifugation the supernatant was stored at 4°C and anti-A titers were measured the following day by adding A erythrocytes in serial plasma dilutions and compared to the original titer. Titers were described as the highest dilution at which hemagglutination was still visible.

We also performed experiments of bacterial incubation with human serum without addition of A erythrocytes, before measuring a possible change in titer the following day. In a parallel experiment, incubation with anti-A plasma took place for 2 hours at room temperature (for IgM binding).

## Results

In the first experiment serum was pre-incubated with unboiled bacterial suspensions in increasing concentrations. After addition of blood group A erythrocytes however, agglutination was still observed. As pre-incubation with viable bacteria did not result in inhibition of hemagglutination, i.e. did not absorb antibodies form the serum, we also measured a possible change in antibody titers. The original titer was diluted threefold with bacterial suspension and A erythrocytes in a 1:1:1 ratio. However, compared to the PBS control, pre-incubation with bacterial suspensions did not lower the titer of anti-A plasma when A erythrocytes were added.

Subsequently bacterial suspensions were boiled for 2.5 hours to unmask antigens that theoretically may have been hidden by the bacterial capsule. IgM titers were reduced but only one-fold, which was regarded as non-significant. When a surplus of bacterial suspension (4,8 × 10e9 cfu/ml) was added, agglutination was still present.

In the last set of experiments, bacterial suspensions were boiled and centrifuged at higher speed (14000 rpm) than the previous experiment to prevent loss of light antigens, or sonicated to prevent denaturation of antigens. Similar amounts of bacterial suspension were incubated with or without A erythrocytes. However, both boiling and sonication did not prevent agglutination compared to PBS control.

## Discussion

We demonstrate a patient with fulminant antibody-mediated rejection after ABO-i kidney transplantation, whose anti-A IgM titers rose dramatically following *S. marcescens* sepsis.

Two features of this post-transplant course are different from other reports of acute rejection in ABO-i kidney transplantation: first, the late occurrence of AMR 12 weeks after kidney transplantation and second, the very high anti-A IgM titers (>5000).

Long term patient survival has not been shown to be significantly different between ABO-compatible and incompatible kidney transplant recipients [10]. However, a higher risk of AMR exists, occurring mainly in the direct postoperative period. In the 51 patients with detailed time of onset of AMR in the literature, only 4 experienced AMR 12 weeks or later after transplantation [11-19]. Of all the 65 recipients of an ABO-i kidney allograft in our center so far, 9 experienced AMR and 3 a combined AMR and cellular rejection, all within three months except for the patient presented in this case report [20]. The pathological role of anti-ABO IgG versus IgM on the ABO-i renal allograft is a matter of debate [7,12,21]. Takahashi hypothesizes on two distinct types of AMR after ABO-i kidney transplantation: he states that type I AMR is caused by re-sensitization due to ABO-blood group antigens, occurs early postoperative and is characterized by an IgG antibody rise. Type II AMR on the contrary is caused by primary sensitization due to ABO-blood group-associated antigens. IgM titer rises more than IgG and it takes longer for this type of AMR to develop [7]. He accompanies this hypothesis with two examples: A 34-year old blood group O recipient receiving a blood group B renal allograft, experienced an AMR on postoperative day 19 during urosepsis with *Klebsiella pneumonia*. IgM rose from 4 to 64 and IgG from < 2 to 4. The second example is a 68-year old male blood group B recipient who received a blood group A renal allograft. On postoperative day 9 his graft was removed because of an untreatable AMR in the presence of Methicillin-resistant *Staphylococcus epidermidis* pneumosepsis. His IgM titer rose from 2 to 512 and IgG remained < 2 during the clinical course. We therefore hypothesized that also this AMR had a different etiology than re-sensitization by blood group A antigens: a Gram-negative sepsis.

ABO antibodies are not ‘natural occurring’ and result from contact with A- and B-like antigens in the intestines via nutrients and bacteria [3,4]. ABO antibodies are either absent at birth or present via placental transfer and breastfeeding. Before the age of 3, the infant’s gut becomes colonized with commensal bacteria expressing A- and B-like antigens. The developing immune system produces antibodies against the antigens not present on its own erythrocytes. The continuing influence of gut bacteria on ABO antibody formation is reflected in permanent detectable IgM titers, for example the IgM titers measured in kidney transplant recipients before ABO-i kidney transplantation. In 1969 Springer and Horton fed 23 very young infants (35 weeks or younger) and 14 adults killed *Escherichia coli* O86 [4]. It concerned blood group A and O individuals, both healthy subjects as well as patients with intestinal disorders: 16 children with diarrhea, 2 adults with ulcerative colitis and 2 adults with colon carcinoma. The majority had a fourfold or greater increase in anti-B antibodies after ingestion, infants more than adults and diarrheic patients more than healthy controls. Moreover, six out of seven infants without a baseline titer had titers of 16 or greater after ingestion. In the same paper, Springer demonstrated that anti-human blood group A and B antibodies in chickens can be neutralized by injecting live *Escherichia coli* O86.

Many Gram-negative bacteria with human blood group activity are identified. For example Yi sequenced the entire Escherichia coli O86 gene cluster and identified all the genes responsible for the blood group B-like antigen biosynthesis [22].

There is more evidence that bacterial suspensions are able to reduce anti-ABO titers by binding these ABO antibodies to the bacteria: Springer et al. assessed the blood group activity of 282 Gram-negative bacteria [9]. Different bacterial suspensions were incubated with series of human serum with minimal 4 agglutination titers for two hours. Almost 50 percent of these 282 strains exhibited anti-ABO activity. Bacteria with only one specificity far outnumbered those with two or all three ABO specificities, in which anti-O and anti-B were predominant.

Strong evidence that gut bacteria are able to trigger ABO antibody formation is reported by Daniel-Johnson et al. [23]. He describes severe hemolytic transfusion reactions in two blood group B recipients of a blood group A platelet donor. Although platelet transfusion is preferably performed ABO identical or at least blood group compatible, the limited availability of matched platelet donors makes platelet donation across ABO barriers a common practice. This is infrequently followed by hemolysis as only a small amount of (ABO-i) donor plasma is present. In contrast, in the by Johnson described blood group A platelet donor the anti-B IgG titer rose to 16384 after taking three tablets of probiotics per day. Furthermore, the solubilized form of this probiotic was found to be able to reduce the measured anti-B in plasma of a randomly chosen blood group A donor threefold, from 64 to 8 after incubation at room temperature *in vitro*.

In ABO-i solid organ transplantation the relation between sepsis and AMR is also reported. A pediatric ABO-i kidney transplant recipient experienced biopsy-proven AMR during pyelonephritis, with an increase in anti-B titers to 64 and 128 for IgG and IgM, respectively [6]. Oya et al. report on an ABO-i living donor liver transplantation with an anti-B titer rise during an intra-abdominal hematoma infected with *Serratia marcescens*. Subsequently thrombotic microangiopathy developed. The authors suggest that interaction between the anti-donor ABO antibodies and the endothelial cells of the graft played a causative role in this microangiopathy [24]. Unfortunately, in our case, we could not demonstrate an anti-A antibody binding capacity of the *S. marcescens* strain isolated from our patient. There are several possible explanations for this. First, the *Serratia* colonies grew rather mucoid which is an indication of a capsule. The capsule might have been impermeable to ABO antibodies. However, even boiling which has been presumed to remove the capsule, or sonication to unmask the bacterial cell wall expressing other antigenic epitopes, did not change the results. Second, the amount of bacteria might have been insufficient. However, we also performed incubation with a very viscous density without a change in titer. Third, assay temperature might play a role. However, temperature was adjusted for IgM antigen binding to room temperature and for IgG antigen binding to 37°C and this did not result in inhibition of agglutination. Fourth, the time for interaction between anti-A antibodies and bacterial epitopes and subsequently with A erythrocytes was shorter than in the experiments carried out by Springer. In addition, Springer and Horton describe in their methods the possibility of ‘non-agglutinating-in-saline’ ABO antibodies and their detection with anti-human serum after immunizing chickens with ABO antigens. We did not explore this possibility.

Next to *S. marcescens* sharing a comparable epitope with antigen A, another explanation for the development of antibody-mediated rejection during *S. marcescens* sepsis exists. This might be a change in antigenicity of the A antigen. *S. marcescens* was cultured in our patient’s urine several times and subsequently in her blood. *S. marcescens* is a Gram-negative bacillus and belongs to the family of *Enterobacteriaceae*[25]. Mannose-sensitive pili of *S. marcescens* are known to stimulate renal scarring [26]. This renal scarring could have hypothetically enhanced changes in ABO antigenicity in the kidney graft.

## Conclusion

ABO antibodies result from contact with gut bacteria. A Gram-negative sepsis could theoretically boost anti-ABO antibody formation. We demonstrated a patient whose anti-A titers rose dramatically after Gram-negative sepsis, leading to a type 3 antibody-mediated rejection. Despite comparable incubation experiments in the literature, we could not demonstrate an interaction between *S. marcescens* and anti-A antibodies. Therefore it remains uncertain whether bacteremia can be the cause of antibody-mediated rejection in ABO-i kidney transplantation.

## Consent

The patient has died. Written informed consent was obtained from the husband for publication of this Case report and any accompanying images. A copy of the written consent is available for review by the Editor of this journal.

## Abbreviations

ABO-i blood group: ABO-incompatible; AMR: Antibody-mediated rejection; cfu: Colony forming units; CMV: Cytomegalovirus; HLA: Human leucocyte antigen; Ig: Immunoglobulin; PBS: Phosphate buffered saline; PCR: Polymerase chain reaction; S. marcescens: *Serratia marcescens* .

## Competing interests

The authors declare that they have no competing interests.

## Authors’ contributions

AdW designed and performed the experiments and wrote the manuscript. AV designed and supervised the experiments and corrected the manuscript. HvdH performed the experiments. MvG supplied the pathology reports and corrected the manuscript. WW treated the patient and corrected the manuscript. MB participated in the literature review and corrected the manuscript. MvA supervises the ABO-i kidney transplant program and corrected the manuscript. All authors read and approved the final manuscript.

## Pre-publication history

The pre-publication history for this paper can be accessed here:

http://www.biomedcentral.com/1471-2369/15/31/prepub
